# DICER1 syndrome and embryonal rhabdomyosarcoma of the cervix: a case report and literature review

**DOI:** 10.3389/fped.2023.1150418

**Published:** 2023-05-05

**Authors:** Alexandre Stambouli, Audrey Cartault, Isabelle Oliver Petit, Solene Evrard, Eliane Mery, Frederique Savagner, Stephanie Trudel

**Affiliations:** ^1^ Molecular Biology Department, Federative Institute of Biology, Toulouse, France; ^2^ Endocrinology Department, Children's Hospital of Toulouse, Toulouse, France; ^3^ Pathology Department, IUCT, Institut Claudius Regaud, Toulouse, France; ^4^ Inserm UMR 1297, Toulouse, France; ^5^ Inserm UMR 1291, CHU Purpan—BP, Toulouse, France

**Keywords:** DICER1 syndrome, cervical embryonal rhabdomyosarcoma, thyroid pathologies, surveillance recommendations, genetic testing

## Abstract

**Background:**

Embryonal rhabdomyosarcomas (ERMS) of the uterine cervix and corpus are rare pediatric tumors usually associated with a late age of onset and frequent somatic DICER1 mutation. It may also develop in the context of a familial predisposition such as DICER1 syndrome requiring specific medical care for children and young adults at risk for a broad range of tumors.

**Case presentation:**

This is a case of a prepubescent 9-year-old girl who was presented to our department for metrorrhagias due to a vaginal cervical mass, initially classified as a müllerian endocervical polyp on negative myogenin immunostaining. The patient subsequently manifested growth retardation (-2DS) and learning disabilities leading to genetic explorations and the identification of a germline pathogenic *DICER1* variant. The family history revealed thyroid diseases in the father, aunt and paternal grandmother before the age of 20.

**Conclusion:**

Rare tumors such as cervical ERMS associated with a family history of thyroid disease during infancy could be related to DICER1 syndrome. Identifying at-risk relatives is challenging but necessary to detect early DICER1 spectrum tumors in young patients.

## Introduction

DICER1 syndrome is a rare autosomal dominant genetic disorder which predisposes to the development of a wide range of both benign and malignant tumors. This syndrome has been linked to pathogenic germline mutations of the *DICER1* gene which encodes an endoribonuclease involved in the maturation of microRNAs (miRNAs) and therefore the regulation of gene expression (1). DICER1 acts as a tumor suppressor where mutations in one gene allele lead to an increased risk of tumor development and the necessity of a somatic mutation in the second allele to produce a malignant phenotype. The majority of germline mutations are located throughout the entire gene, whereas somatic mutations have been found in the regions that encode the RNase III domains.

DICER1 syndrome is strongly suspected when typical DICER1-associated tumors such as lung cysts, pleuropulmonary blastoma (PPB), cystic nephroma, ovarian sex-cord stromal tumor and multinodular goiter develop in the early stages of childhood (2, 3). Recently a list of less commonly observed tumors including embryonal rhabdomyosarcoma (ERMS) of the bladder, uterine cervix or fallopian tubes have also been described (4–6).

Incomplete penetrance of germline pathogenic *DICER1* variant and the low risk for relatives of developing a tumor before the age of 10 complicate care recommendations which have been recently discussed (7). Although DICER1 syndrome remains a rare entity it often leads to a delay in medical care for the proband with a high risk of malignant tumor development.

This is a case report of a female child incidentally diagnosed with rare *DICER1*-associated ERMS of the cervix after multiple investigations.

## Case report

The female patient was born at term with a history of intrauterine growth restriction (IUGR; s:46 cm; w:2,550 g, hc: 34 cm) and neonatal dysmorphia, leading to neonatal urinary glycosaminoglycans (GAGs) analysis for screening of mucopolysaccharidosis with inconclusive results. She is a first child born at 39 weeks of amenorrhoea after normal pregnancy and delivery (mother's age: 24 yrs). At the age of 9 the girl was referred to our pediatric gynecology department for metrorrhagias. Clinical and physical examination revealed no sign of pubertal development but retrieved a cervically protruding vaginal mass corresponding to multiple polyps of 0.5 to 2 cm length. After surgical resection, the histopathological analysis indicated benign müllerian endocervical polyps with variable cellularity, mild atypia, squamous-like epithelium and diffuse Smooth Muscle Actin but negative Myogenin immunostaining. Serum tumor markers alpha-fetoprotein (AFP), carcinoembryonic antigen (CEA) and human chorionic gonadotropin (hCG) were negative.

**Figure 1:**
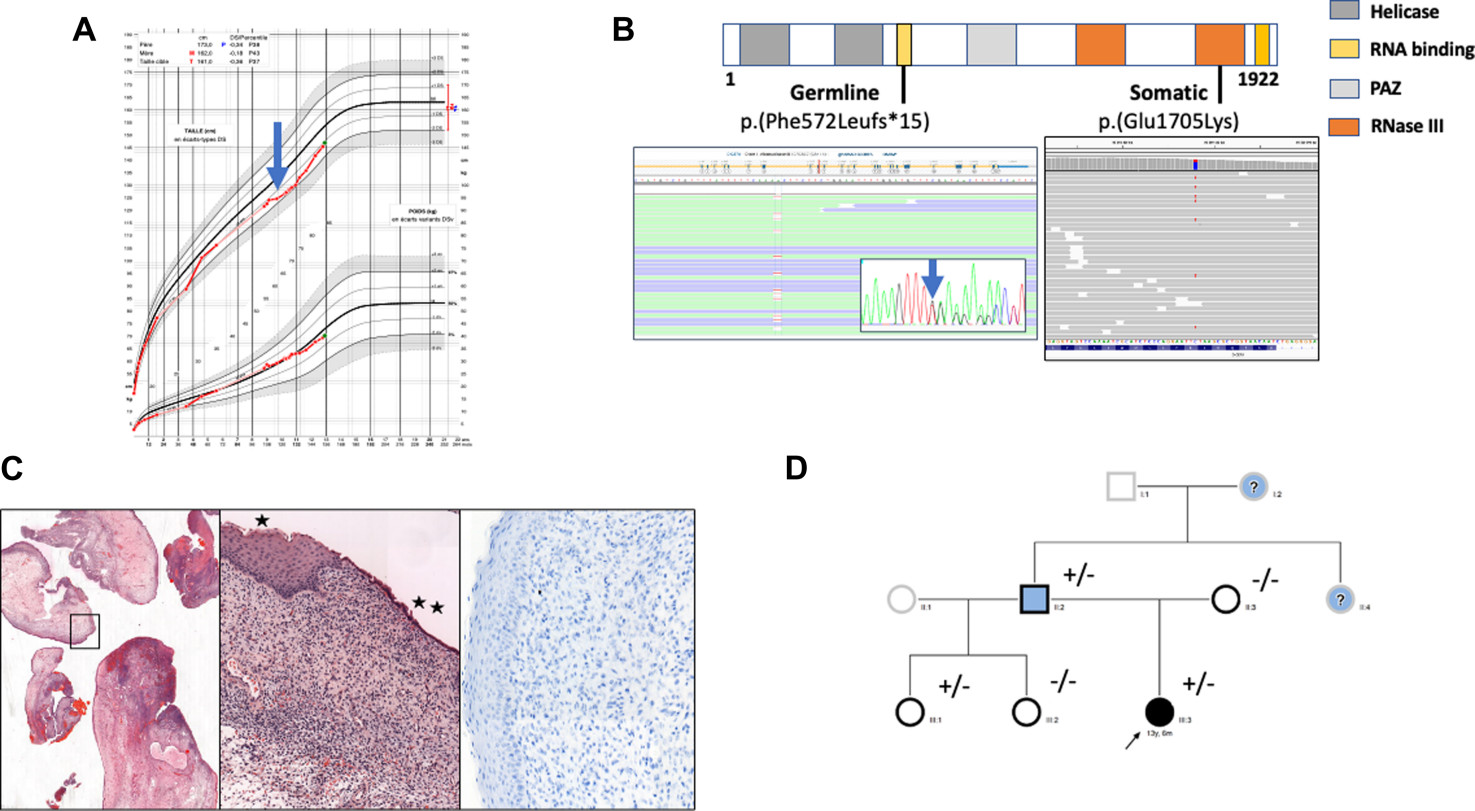
(**A**) growth curve of the female child. At 10 years old growth retardation (-2 SD) was noted (blue arrow) and synthetic growth hormone treatment was started. (**B**) Localization of identified somatic and germline variants. Germline heterozygous T deletion (c.1716del, p.(Phe572LeufsTer15)) and somatic heterozygous substitution c.5113G > A, p.(Glu1705Lys) were detected by next generation sequencing (Illumina, custom metabolic disorders panel) in exon 10 and 27 respectively of the *DICER1* gene (LRG_492, NM_177438.3; Sequencing depth was 874 x  and 756 x  respectively (Integrative Genomic Viewer Software). The germline variant was confirmed by Sanger sequencing (blue arrow). (**C**) Histologic and immunohistochemical examination of cervical tissue samples. Edematous congestive polyp fragments, hematoxylin and eosin (HE) staining, original magnification×25 (left panel); Surface squamous (*) or mucinous (**) epithelium without atypia, the underlaying proliferative cells are monotonous without atypia or mitosis, HE×100 (middle panel); Myogenin staining showing no myogenin expression,×200 (right panel). (**D**) Family pedigree. Individuals screened for *DICER1* germline variant are indicated by a black border. Clinical phenotypes are represented by black filling for ERMS, blue for thyroid disease and white for asymptomatic. The proband is marked with an arrow. “+” and “-” indicate the mutation status. Question marks correspond to symptomatic patients whose *DICER1* mutational status is unknown.

A year later, during clinical follow-up, the patient presented a decline in growth rate with growth retardation (-2 SD) (Figure 1A). A physical examination revealed prognathism, macroglossia and brachymetacarpia with no other remarkable factors. Isolated partial growth hormone (GH) deficiency was diagnosed on stimulation tests. Imaging tests, including hand and wrist x-ray, thyroid echography and pituitary magnetic resonance imaging (MRI) were all normal. Further cytogenetic and molecular testing showed normal karyotype (46XX) and no *SHOX* deficiency. Complementary hormonal results ([thyroid stimulating hormone (TSH), free thyroxine (FT4), parathyroid hormone (PTH), adrenocorticotropic hormone (ACTH), cortisol, prolactin, estradiol, anti-müllerian hormone (AMH), insulin-like growth factor 1 (IGF1), insulin-like growth factor binding protein-3 (IGF-BP3)]) before synthetic GH treatment (initial dose: 0.3 mg/kg/week) were all normal (details in Table 1).

**Table 1:** Clinical and hormonl features for the index case and her mother.

			**Reference range**
**Maternal features**			
Age (yrs)	24		
Size (cm)	162		
**Neonatal features**			
Size (cm)	46		
Weight (g)	2.551		
Head Circonference (cm)	34		
At 9 yrs		At 12 yrs	
Before GH treatment		During GH treatment	
TSH	1.6	1.3	0.5–5 µIU/ml
FT4	14.7	14.3	8.6–25 pg/ml
PTH	22		6–50 pg/ml
ACTH	16		10–50 pg/ml
Cortisol 8 h	5.88		5–23 µg/dl
Prolactin	169		85–325 µUI/ml
Estradiol	23	22	< 35 ng/L
AMH	1.3		0.2–2.8 ng/ml
IGF	191	436	111–990 ng/ml
IGF-BP3	4.05	4.99	2.4–10 mg/L
**LHRH test**			
LH peak (mIU/ml)	2.1		
FSH peak (mIU/ml)	8.7		
**At 10 yrs—before GH treatment**			
Clonidine test- GH peak (ng/ml)	6		
L-DOPA test -GH Peak (ng/ml)	2.4		
Bone age (hand and wrist x-ray)	9 years 10 months		

At the age of 12, due to scholastic difficulties and learning disabilities associated with facial dysmorphism and a history of screening for mucoploysaccharidosis, a more comprehensive genetic exploration was performed using a broad gene panel for rare pediatric disorders combined with next-generation sequencing (NGS)(for complete panel see additional Supplementary Material Table S1). After obtaining written informed consent for genetic testing, no variant in mucopolysaccharidosis-related genes could be identified while an heterozygous pathogenic frameshift variant in exon 10 of the *DICER1* gene (NM_177438.3) designated as c.1716del; p.(Phe572LeufsTer15) was found. This variant leads to a premature stop codon in the P-loop containing the nucleoside triphosphate hydrolase-domain of the protein. The mutation was confirmed on a second blood sample.

The identification of this *DICER1* germline mutation led us to perform a molecular analysis on the endocervical mass. We found a second-hit somatic missense variation on the other *DICER1* gene allele designated c.5113G > A; p.(Glu1705Lys) in exon 24 (allele frequency: 27%) corresponding to the known genetic hotspot for somatic mutations in the ribonuclease III domain (Figure 1B).

In light of *DICER1* gene germline and somatic mutations, a pathological reassessment of the cervical resection was performed in a reference center, allowing reclassification from benign müllerian polyps to embryonal rhabdomyosarcoma with immunostaining focally positive for desmin and still negative for myogenin and myoD1 (Figure 1C). Together, all these results led to a diagnosis of DICER1 syndrome and the appropriate medical care for the patient and her family.

Family screening revealed that the father carried the same nucleotide deletion, which is consistent with thyroidectomy at the age of 16 for a multinodular goiter. A history of benign thyroid nodules in the father's mother and sister was also reported (Figure 1D). No other DICER1-related pathologies have been described in the family especially no PPB over three generations. Furthermore, extended genetic counseling was suggested for the father's family, in particular the proband's half-sisters. The youngest, who was 2 years old, carried the same familial deletion but presented no clinical or imaging manifestations at screening.

## Discussion

DICER1 syndrome is associated with a panel of benign and malignant tumoral manifestations, which, in terms of unusual phenotypes, has not yet been completed. Our case is consistent with a double-hit tumorigenic process: the germline pathogenic variant being a frameshift caused by a deletion and the somatic variant found in the cervical tumor being a missense mutation c.5113G > A; p.(Glu1705Lys) in the catalytic domain of the protein, previously described in DICER1-related tumors (8). Concerning the germline variant c. 1716del, it was previously described in an infant with a PPB at the age of 0.8 years, corresponding to the youngest child in the series of 11 PPB-families (9).

ERMS is the most common subtype of rhabdomyosarcoma, with a high prevalence in the head and neck region as well as in the genitourinary tract, usually characterized by a positive immunohistochemistry for desmin, myogenin and MyoD1 (10, 11). The “botryoid” variant is a particular form of ERMS found in mucosa, typically presenting as a polyp. However, ERMS of the uterine cervix and corpus are particularly rare affections and, in our case the primary analysis showed no such typical immunostaining profile, which led to an inaccurate diagnosis of benign müllerian polyps. Moreover, *DICER1*-associated ERMS occurs most frequently in pubertal and post-pubertal adolescent girls and young women (mean 10–20 years). A recent study including five cervical and four uterine ERMS showed *DICER1* pathogenic variants in all the uterine lesions whereas these variants were absent in the urinary tract ERMS. The authors suggest that *DICER1*-associated ERMS could be qualified as a distinct subtype in future classifications, especially when related to a specific DNA methylation profile (12).

Growth retardation was an important feature in this case, but is not usually related to DICER1 syndrome. In their exploration of pituitary development in families with the *DICER1*-truncating variant *Zhang* et al*.* showed that changes in miRNAs level could be the cause of clinical pituitary disorders but no evidence of a direct relation with growth hormone deficiency was noted (13). However, in a recent study on unusual symptoms of the DICER1 syndrome, *Venger* et al*.* described developmental delay and facial dysmorphism in a family carrying a *DICER1* frameshift variant (6). Considering the higher risk of cancer development for pediatric patients receiving GH treatment, this young girl carrying a germline *DICER1* pathogenic variant should benefit from reinforced sureveillance relative to current guidelines (14).

Family history is an important aspect in evaluating the risk of DICER1 syndrome. In this case, the association of a childhood tumor, although initially considered to be benign, and a familial history of multinodular goiter should alert the clinician to initiate genetic testing for *DICER1* (ideally included in a NGS gene panel). In addition, screening and surveillance recommendations differ based on age and gender. Therefore, chest imaging is recommended from birth until 12 years of age since PPB is the main lung expression of DICER1 syndrome, whereas thyroid and pelvic ultrasound (US) are only recommended as of 8 years of age throughout life (7, 15). For this young girl who became pubertal in accordance with the standard timelines, follow-up was initiated and included regular physical exams, yearly pelvic and abdominal US until the age of 40, and thyroid US every 3 years. Fine needle aspiration biopsy (FNAB) could be used when focal changes in the thyroid gland appeared but due to the 16 to 18-fold increased risk of differentiated thyroid cancer especially in female *DICER1* carrier, thyroidiectomy should be rapidly considered (16, 17). This was also the case for all the relatives' female children who were carriers of *DICER1* deletion. The initial treatment was surgical resection alone (no additional treatments), and four years later any local or distant recurrences were monitored.

This case report highlights that DICER1 syndrome should be suspected in the presence of early typical DICER1-related tumors as well as in case of unusual tumors during childhood, especially when there is a family history of thyroid diseases during infancy or young adulthood.

## Data Availability

The datasets presented in this article are not readily available because of ethical and privacy restrictions. Requests to access the datasets should be directed to the corresponding author.
